# Actinomycetoma by Nocardia asteroides: A Case Report of a Unique Neglected Tropical Disease From North India

**DOI:** 10.7759/cureus.57753

**Published:** 2024-04-07

**Authors:** Sweta Singh, Arijit Jotdar, Niraj Kumari, Sana Islahi, Shefali Gupta

**Affiliations:** 1 Clinical Microbiology, All India Institute of Medical Sciences, Raebareli, IND; 2 Otolaryngology - Head and Neck Surgery, All India Institute of Medical Sciences, Raebareli, IND; 3 Pathology and Laboratory Medicine, All India Institute of Medical Sciences, Raebareli, IND; 4 Microbiology, All India Institute of Medical Sciences, Raebareli, IND

**Keywords:** tumefaction, filamentous, tropical, neglected, mycetoma, nocardial

## Abstract

Nocardial mycetoma is a neglected tropical disease reported worldwide, especially in tropical and subtropical regions. It is ubiquitous in nature and is a soil-borne, gram-positive, filamentous, aerobic bacteria with acute angle branching. Traumatic inoculation in endemic areas is the primary mode of infection of this debilitating disease. The clinical triad of tumefaction, draining sinus, and pus discharge with granules is very much characteristic and specific for clinching the diagnosis of mycetoma. However, the painless nature of the primary skin lesion often makes the patient present late to the clinician, often in the advanced stages of the disease. Here, we present a very intriguing case report of a young female patient who presented with a single neck nodule but was later diagnosed as a case of nocardial mycetoma. Timely diagnosis and initiation of therapy proved to be a boon for the patient with almost complete recovery within a few weeks in the form of healed skin lesions and insignificant scarring.

## Introduction

*Nocardia* infections are on the rise in today's developing world. The cases of nocardiosis are prevalent worldwide with substantial contributions from developing countries. It is a challenging infection in the form of chronic granulomatous subcutaneous inflammatory disease and has a significant impact on social and medical aspects [1,2]. Mycetoma is a clinical entity that can be both bacterial (actinomycetomas) and fungal (eumycetomas) in origin. The cases of mycetomas are endemic in several tropical and subtropical countries. The mode of transmission of the infection is by traumatic inoculation as the causative pathogen is a natural inhabitant of soil, decaying wood, water, and air [3,4]. This later gives rise to an acute inflammatory reaction, which results in abscess formation with necrosis and discharge of characteristic granules or granuloma formation in some cases. The clinical triad of tumefaction, draining sinus, and pus discharge with granules is very much characteristic and specific for clinching the diagnosis of mycetoma in patients. Cutaneous involvement in the cases of nocardiosis may present as either of the following forms: (1) acute superficial skin infection with abscesses or cellulitis, (2) mycetoma, (3) lymphocutaneous (sporotrichoid) infection, or (4) disseminated infection with skin involvement [1,3].

*Nocardia* is very unique in its genus characteristics as it is a gram-positive, aerobic, acid-fast, filamentous bacteria with branching at right angles, often fragmenting into bacillary and coccoid forms. Various species of *Nocardia* have been identified so far as pathogens of humans, namely,* Nocardia asteroides* complex, *Nocardia brasiliensis*,* Nocardia farcinica*, and *Nocardia​​​​​​​ nova* [1]. *Nocardia​​​​​ brasiliensis *are usually implicated in subcutaneous infection, while *N. asteroides* cause more serious invasive infection presenting commonly as pneumonia in patients who are immunocompromised, have underlying chronic lung disease, or are on long-term steroid therapy [2,3].

Laboratory diagnosis of *Nocardia* in mycetoma cases involves the collection of granules from the discharging pus, followed by gram and acid-fast staining. They are usually appreciated as gram-positive, filamentous, acid-fast bacteria with right angle branching. Isolation on culture is usually done on routine media such as blood agar, Lowenstein Jensen (LJ) media, and Sabouraud's dextrose agar (SDA). Growth on culture followed by various biochemical reactions or molecular tests can provide a definitive diagnosis in mycetoma cases [5,6].

Prompt diagnosis and early initiation of chemotherapy in cases of nocardial mycetoma can show dramatic improvement and negate the possibility of surgical measures such as deep tissue debridement or amputation. This not only results in improving the quality of life of the patient but also has a positive impact on one's social and personal life. Mycetoma often comes in the list of neglected tropical diseases as the clinical diagnosis is often delayed due to a lack of suspicion on the part of treating physicians, its varied clinical presentations, and its similarity with fungal infections such as deep mycotic ones.

Here, we present an intriguing case report of a young female belonging to a rural area who presented to our setting with only a complaint of a neck nodule but was later diagnosed as a case of mycetoma with multiple sinuses and abscesses all over the body. Prompt diagnosis and early initiation of chemotherapy proved to be a boon, which resulted in the resolution of the infection within three months.

## Case presentation

**Figure 1:**
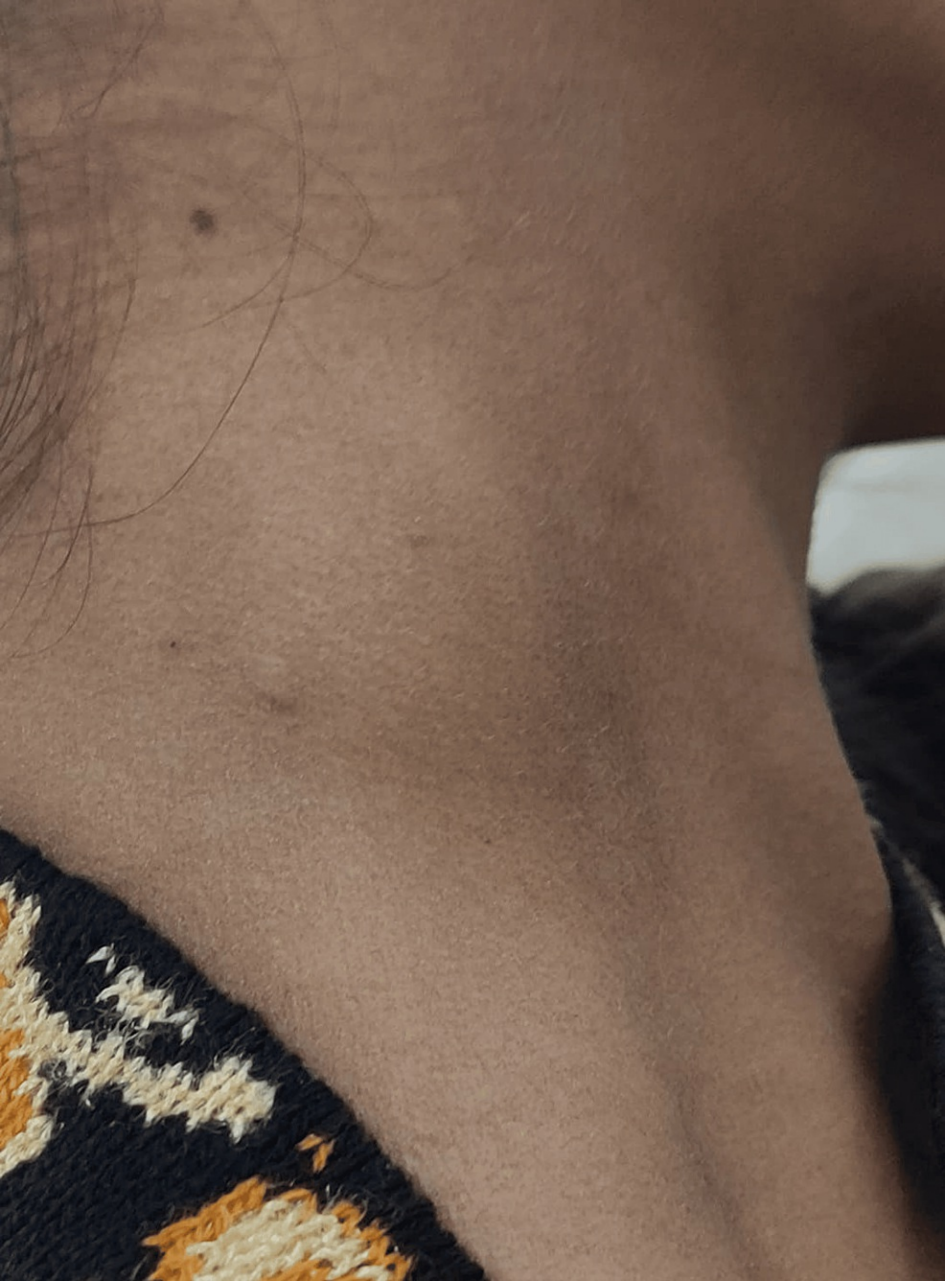
Clinical image of the patient with neck nodule (front view)

**Figure 2:**
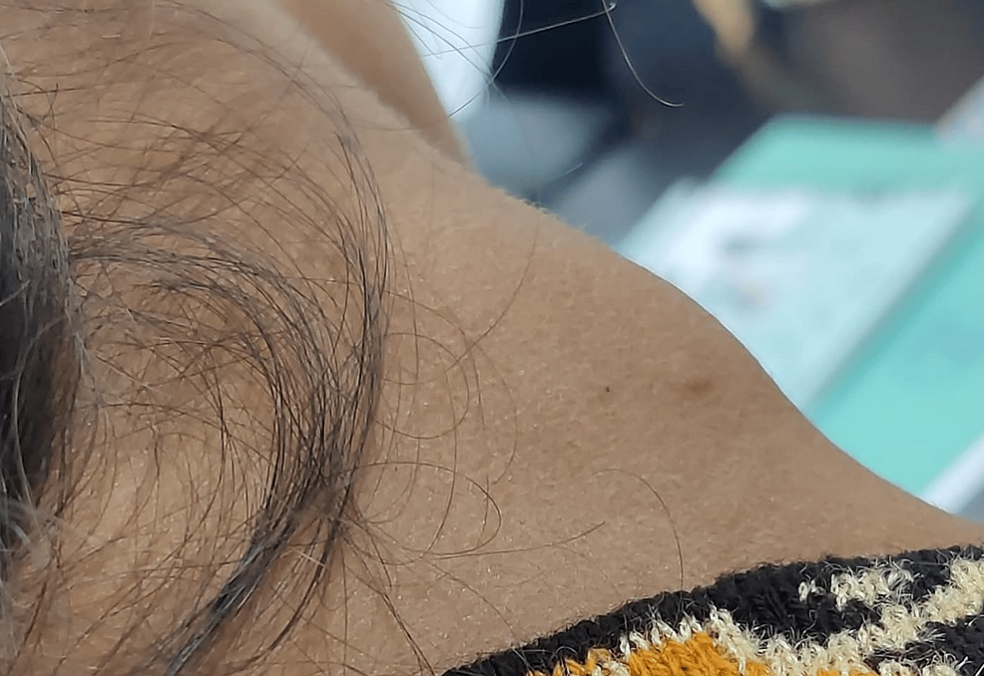
Clinical image of the patient with neck nodule (lateral view)

A 17-year-old female laborer presented to the outpatient department of otolaryngology with a chief complaint of pain and swelling over the right neck region for the last three months (Figures 1, 2). She gave the present history of progressive increase in the size of the swelling over the past two months with slight pain and redness. According to her, the swelling was initially painless, but in time, it developed pain and tenderness. There was no increase in the number of swellings and no associated dysphagia or odynophagia. There was no significant history of any fever, rash, upper respiratory tract infection, weight loss, myalgia, or any chronic disease. She had neither any recent travel history nor any contact with animals or pets. She used to work at construction sites as a brick carrier for the last year.

**Figure 3:**
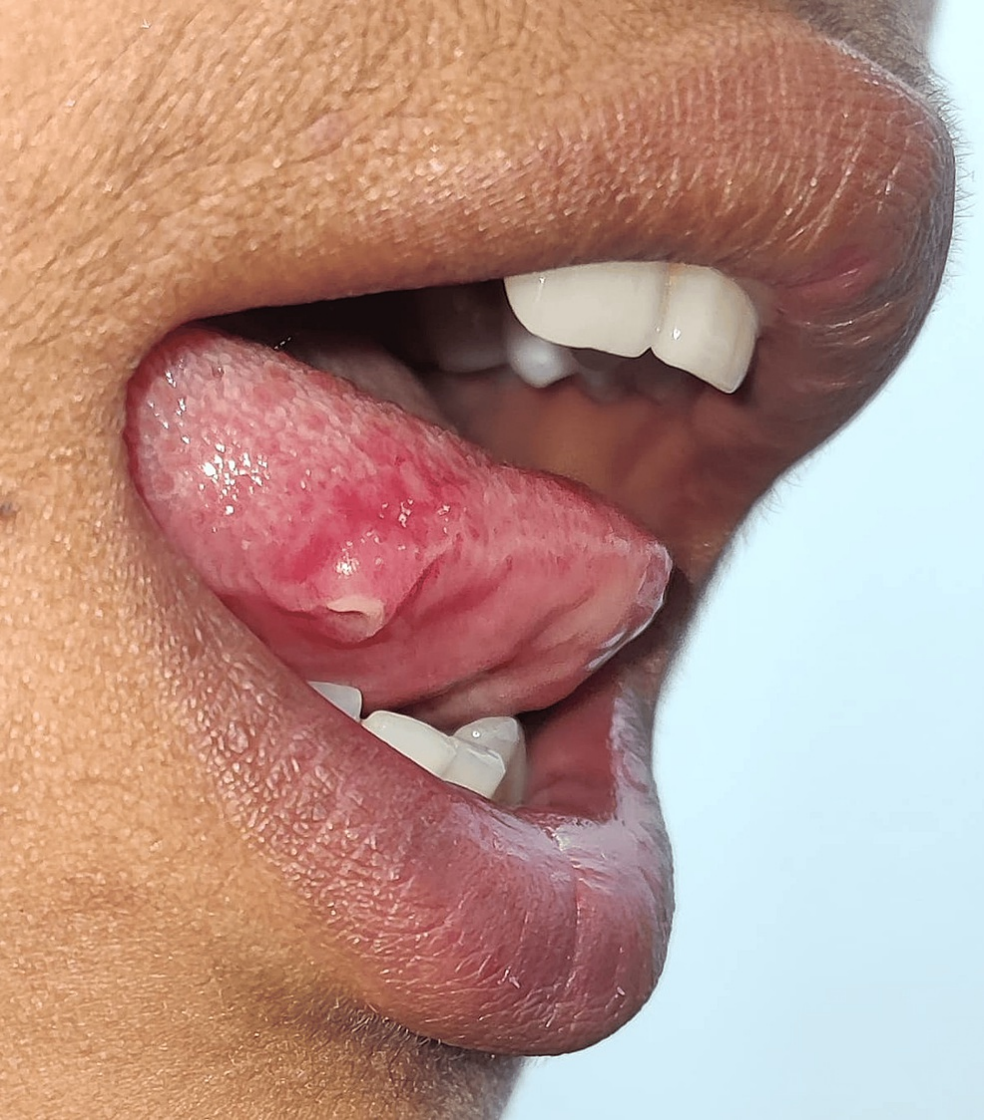
Clinical image of the patient with lesions in the oral cavity

**Figure 4:**
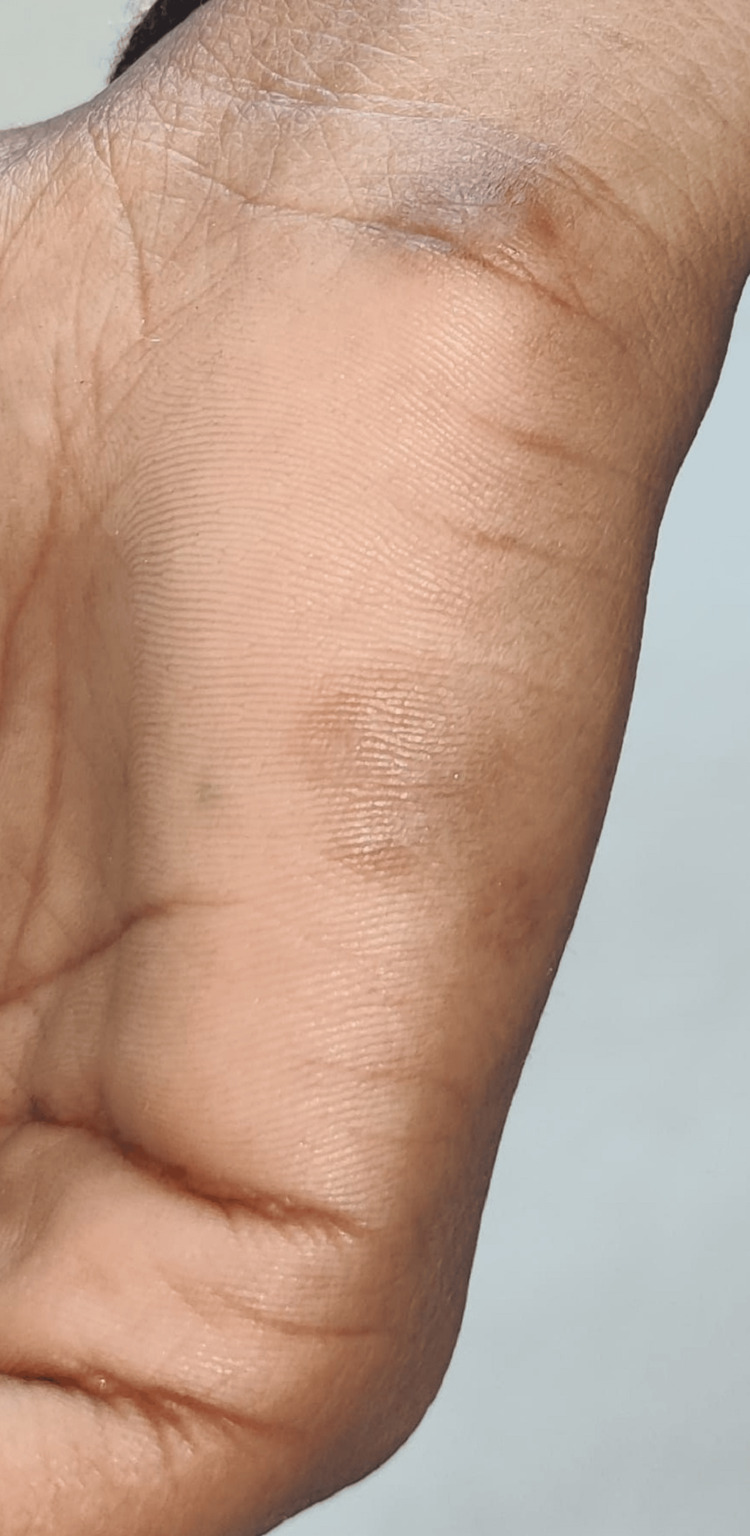
Clinical image of the patient with lesions on the hand and palm

**Figure 5:**
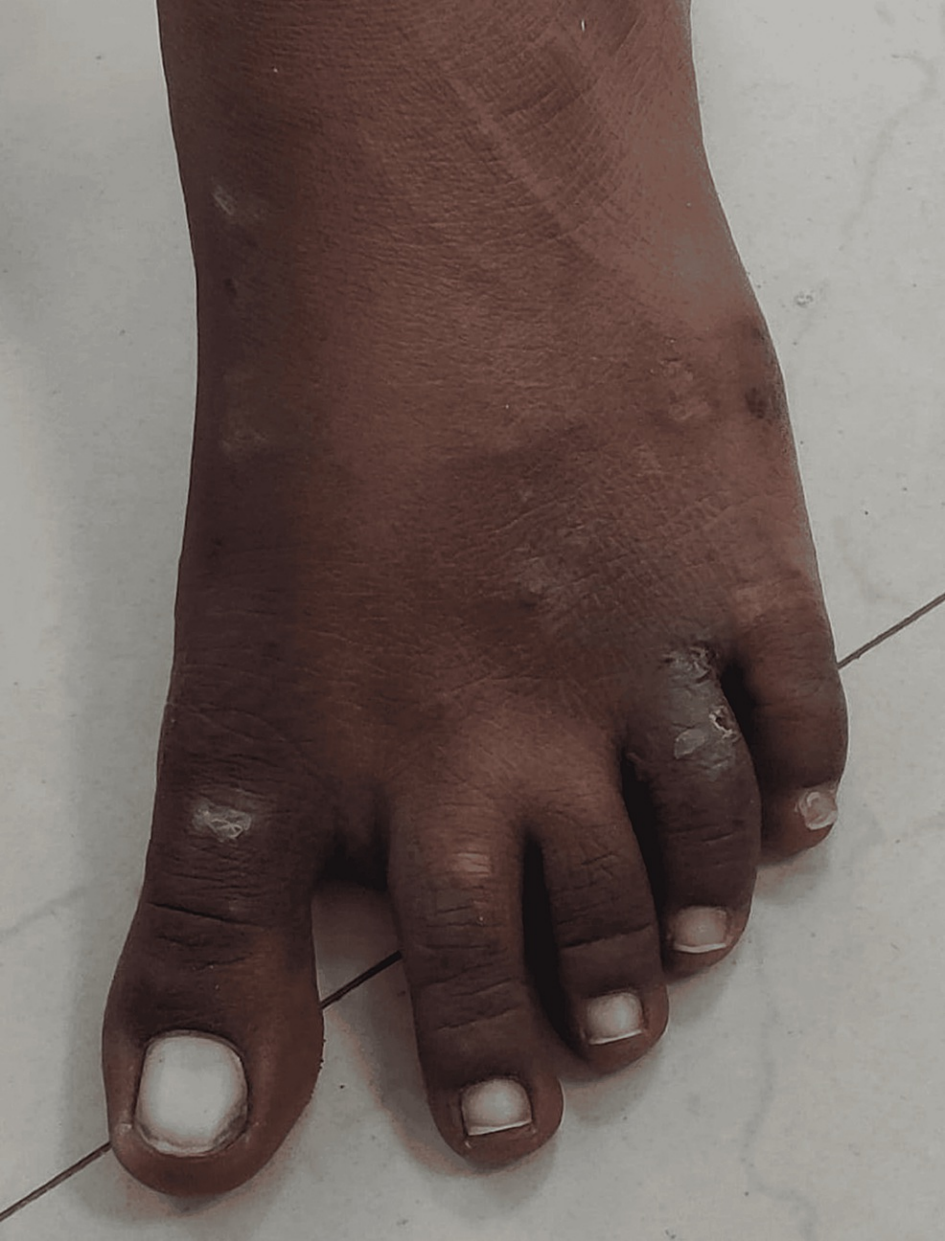
Clinical image of the patient with lesions on the foot and toes

On clinical examination, the clinician examined multiple healed and unhealed lesions on the oral cavity, right hand, and both lower limbs (Figures 3-5). The lesions on the lower extremities were present in the thigh and toe region with discharging pus. However, the pus was scanty in amount and with no specific odor or granules. The lesions on the hand were dry, healed, and with no pus discharge. On further enquiring, the patient also revealed a history of development of on and off mouth ulcers on the base and lateral area of the tongue with no pus discharge (Figure 3).

History revealed that she had initially developed a few pruritic papule-like lesions on the toes of the right lower limb, which later on became erythematous with seropurulent discharge that was granular in consistency. The patient recalled a minor trauma at the construction site while carrying bricks seven months back. The swelling on the neck region was warm to the touch, indurated, mildly tender, and had a boggy feel with no associated cervical lymphadenopathy.

Other findings from her systemic examination and routine investigations, including chest X-ray, were normal. X-ray films of the involved right neck region showed soft tissue swelling suggestive of thickened overlying skin and exudate collection in fascial planes.

**Figure 6:**
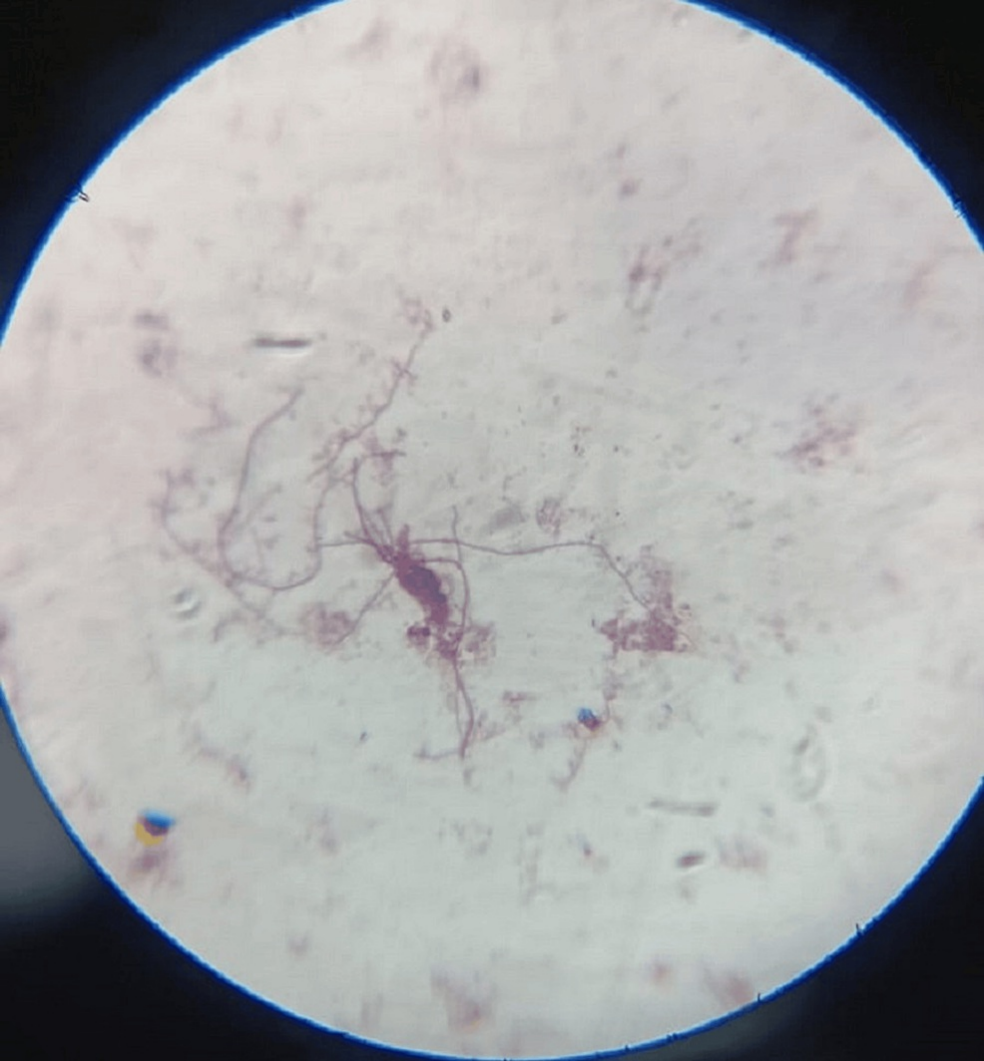
Nocardia on microscopic examination (gram stain)

**Figure 7:**
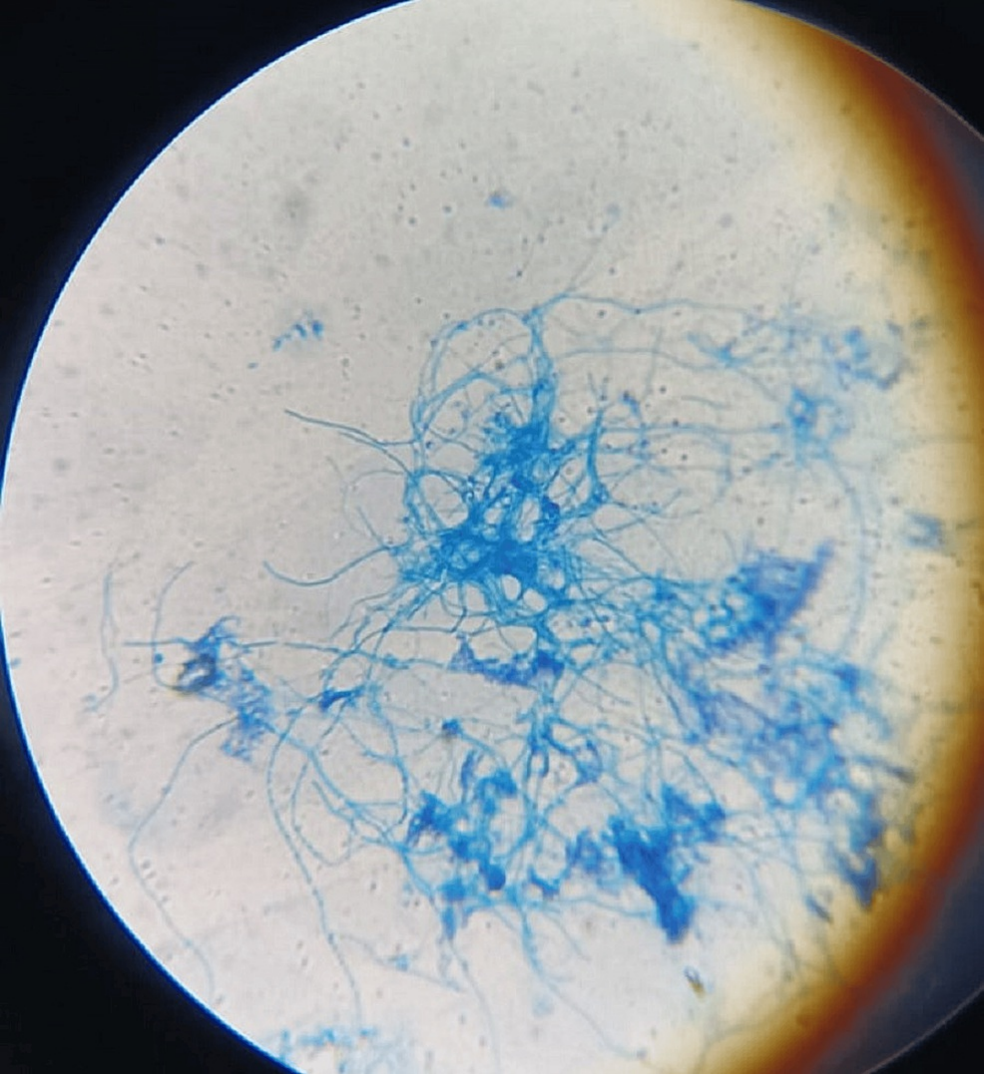
Nocardia on microscopic examination (modified ZN stain) ZN: Ziehl-Neelsen

**Figure 8:**
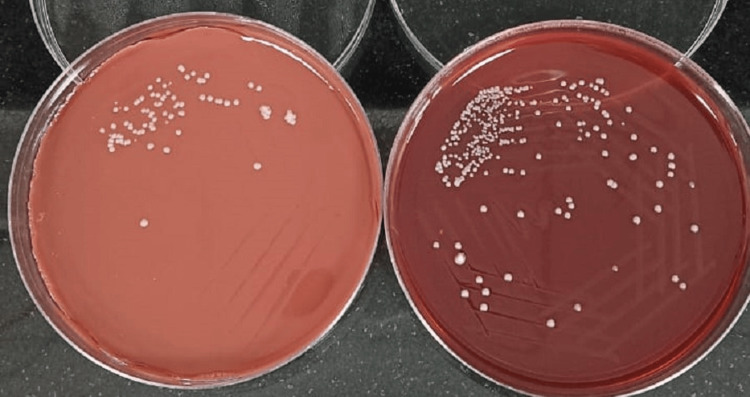
Nocardia colony morphology on chocolate agar (left) and blood agar (right)

**Figure 9:**
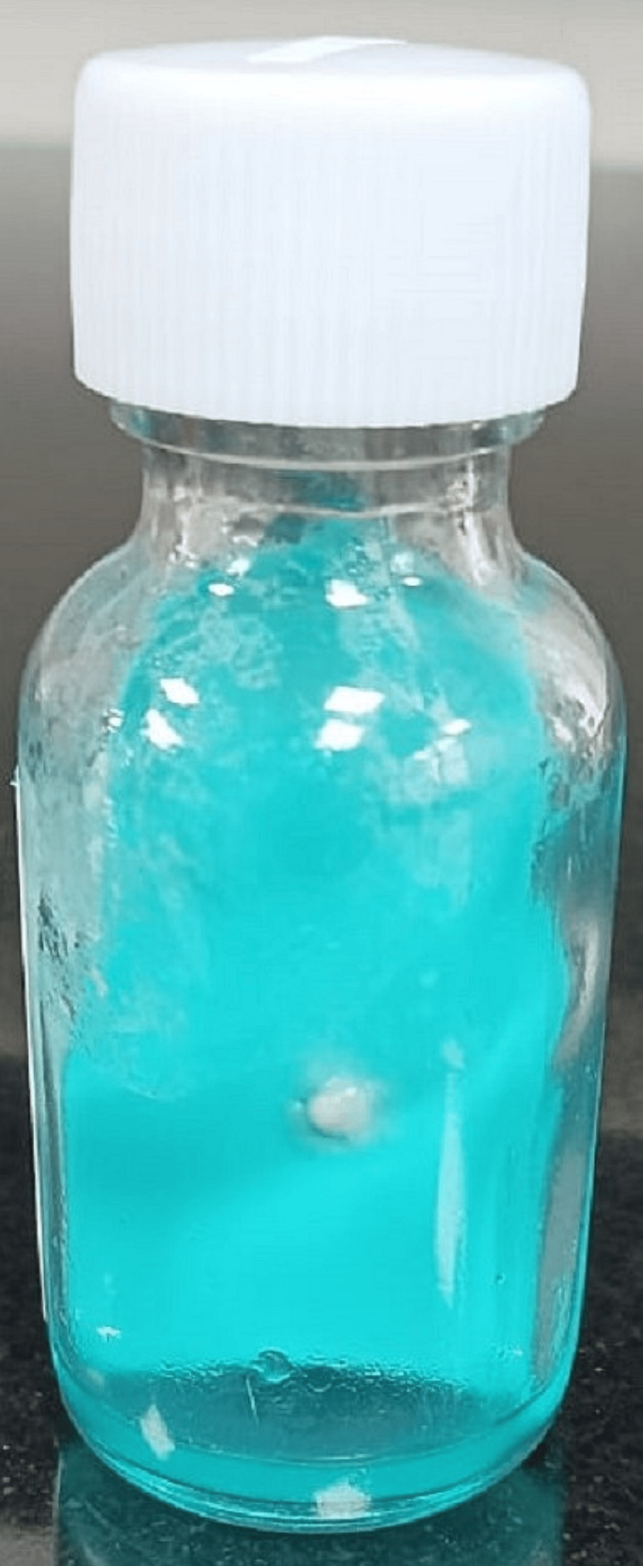
Typical growth of Nocardia colonies on LJ media LJ: Lowenstein Jensen

**Figure 10:**
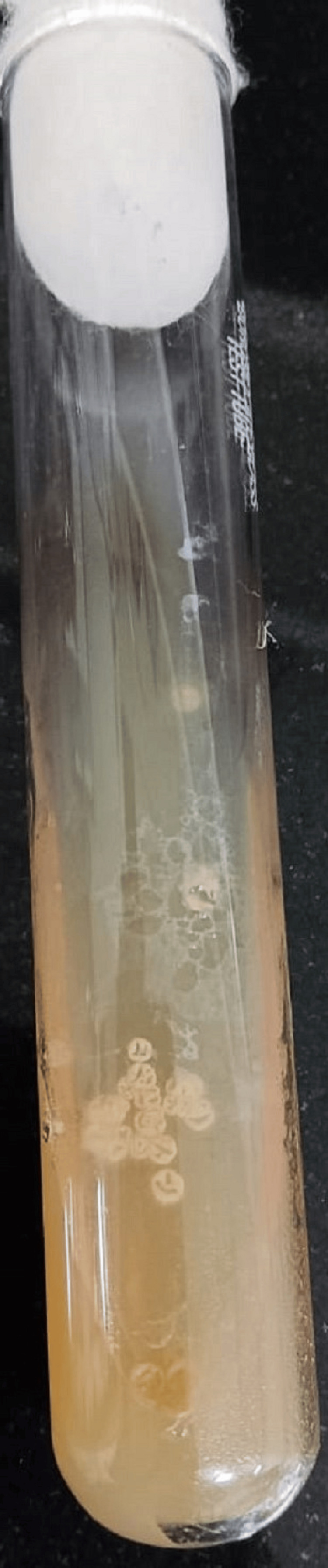
Typical growth of Nocardia colonies on SDA SDA: Sabouraud's dextrose agar

The fine needle aspiration cytology (FNAC) sample from the neck region along with pus from the toe region were sent for microbiological investigations and culture. Gram stain of both samples yielded multiple gram-positive filaments with right angle branching (Figure 6). Further, modified Ziehl-Neelsen (ZN) staining revealed the structures to be acid-fast, thus raising the clinical suspicion of *Nocardia* infection (Figure 7). Cultures were put up in blood agar, chocolate agar (Figure 8), Lowenstein Jensen (LJ) media (Figure 9), and Sabouraud's dextrose agar (SDA) (Figure 10) and incubated overnight. Typical growth of *Nocardia* colonies was seen on all media within four days of incubation appearing as chalky white, cotton candy. Further, gram and modified ZN staining confirmed the probability of *Nocardia* species as gram-positive, acid-fast, filamentous bacteria with right angle branching.

The colonies were further subjected to species identification using various biochemical tests such as urease, casein, xanthine, nitrate reductase, and hypoxanthine to see the hydrolysis of media, utilization of acetamide, citrate, L-rhamnose, and D-sorbitol. Based on the cultural, morphological, and biochemical reactions, the isolate was identified as *Nocardia asteroides*. Histopathology smears from right cervical (level III) lymph nodes showed well-formed granulomas along with multinucleated giant cells. The background showed a polymorphous population of lymphoid cells admixed with blood, suggestive of granulomatous lymphadenitis.

Antibiotic susceptibility testing was further done by E-test and broth microdilution, and the zone diameters were interpreted according to Clinical and Laboratory Standards Institute (CLSI) guidelines. The isolate was found sensitive to aminoglycosides (gentamicin and amikacin), trimethoprim-sulfamethoxazole (TMP-SMX), minocycline, and linezolid and was resistant to ceftriaxone and other cephalosporins. The patient was started on co-trimoxazole (sulfamethoxazole 800 mg + trimethoprim 160 mg bid) and dapsone (100 mg/day) with an additional course of amikacin for five weeks. This specific treatment, according to the patient's condition, has been chosen according to the sensitivity pattern of the isolate based on local antibiogram data. Co-trimoxazole with dapsone and amikacin has shown favorable outcomes in similar cases according to previous studies and case reports. Significant improvement in the toe and hand lesions was noted with healing of the discharging sinuses and puckered scarring within two months. The patient was advised further follow-up after a month in the outpatient department (OPD).

## Discussion

Infections by *Nocardia* species are reported throughout the world with maximum cases reported from tropical and subtropical countries. The cases of actinomycetoma are more often reported in subtropical regions, particularly in America (Mexico and Venezuela), whereas eumycetoma predominates in developing countries such as Africa and India [7,8]. The spectrum of infections by *Nocardia* species is not only limited to immunocompromised individuals but is also seen in immunocompetent ones, although immunocompromised states such as malignancies, immunosuppression due to drugs or antimetabolites, steroid intake, and chronic diseases pose a higher risk of acquiring nocardial infections [9]. The pulmonary system is the most common site of involvement seen in approximately 71% of cases. Further, dissemination is seen in secondary sites such as the brain (42%), spinal cord (24%), chest wall, pleural cavity (8%), skin, and subcutaneous tissue (8%) [9,10]. The differential diagnosis for nocardiosis ranges from tuberculosis to pneumonia, histoplasmosis, bacterial abscess, and lung malignancies. Mortality rates are much higher for immunocompromised and disseminated cases.

Subcutaneous lesions of *Nocardia* usually manifest as mycetoma in immunocompetent cases (7%), and *N. asteroids* is implicated in the majority of them as was seen in our case study [11]. As a soil saprophyte and because of its ubiquitous nature, traumatic inoculation from the soil is the major route of transmission of such infection. Walking barefoot and working outdoors at construction sites or crowded rural areas are the major risk factors [11]. A similar scenario was seen with our patient who worked as a laborer at a construction site. The lesion at the primary site of inoculation starts with a simple abscess, progressing to cellulitis and discharging multiple sinuses in the course of dissemination. The development of mycetoma depends on a few factors such as inoculum size, immune status of the patient, and hormonal adaptation [11-13].

Regarding the rates of progression in mycetoma cases, grave consequences in the form of joint mutilation and loss of function are frequently seen in actinomycotic ones, and bone involvement is relatively rare [13]. In most cases, subcutaneous and cutaneous involvement in the *Nocardia* cases is responsive to chemotherapy. The first-line drugs in such patients are trimethoprim-sulfamethoxazole (TMP-SMX), dapsone, minocycline, and linezolid, along with aminoglycosides such as gentamicin and amikacin [13,14]. Our patient also showed a good response with a combination of co-trimoxazole (sulfamethoxazole 800 mg + trimethoprim 160 mg bid) and dapsone (100 mg/day) with an additional course of amikacin. Symptomatic relief along with healing of lesions was noticed in less than three months.

Mycetoma is a very distinct infection with characteristic clinical features on presentation to the treating physician or clinician. The patient in our case presented to the outpatient department with the only complaint of a neck nodule but was later diagnosed as a case of mycetoma based on a detailed history and clinical examination. This signifies the importance of proper history-taking and clinical examination in such cases of subcutaneous nocardiosis. Also, proper correlation is required between pathology and microbiology reports of the biopsy and FNAC samples of the suspected patients [14]. The differential diagnosis of mycetoma is an exhaustive list that not only includes thorn and foreign body granulomas, particularly in mycetoma endemic areas [15], but also includes many soft tissue tumors such as Kaposi's sarcoma, fibroma, malignant melanoma, or fibrolipoma, keloids, and dermatological conditions such as botryomycosis, sporotrichosis, and plantar or acral psoriasis [16,17]. Therefore, a high index of suspicion is required on the part of the treating clinician, pathologist, and microbiologist to establish a timely diagnosis and initiate therapy in such cases for a favorable outcome.

## Conclusions

Mycetoma is a chronic debilitating neglected tropical disease of the tropical and subtropical areas. It has an immense negative impact on the medical, socioeconomic, and health aspects of patients in endemic areas. Further, as mycetoma lesions are usually painless, patients usually present late with an advanced stage of the disease. Early diagnosis and prompt initiation of chemotherapy are the key factors in stopping the deleterious effects of this disease and improving the quality of life of patients.

## References

[1] Bonifaz A, Tirado-Sánchez A, Calderón L (2014). Mycetoma: experience of 482 cases in a single center in Mexico. PLoS Negl Trop Dis.

[2] Ahmed AO, van Leeuwen W, Fahal A, van de Sande W, Verbrugh H, van Belkum A (2004). Mycetoma caused by Madurella mycetomatis: a neglected infectious burden. Lancet Infect Dis.

[3] Fahal AH, Bakhiet SM (2023). Mycetoma and the environment. PLoS Negl Trop Dis.

[4] Williams NS, Bulstrode C, O'Connell PR (2013). Bailey and Love's short practice of surgery, 26th edition. http://pp..

[5] Maraki S, Chochlidakis S, Nioti E, Tselentis Y (2004). Primary lymphocutaneous nocardiosis in an immunocompetent patient. Ann Clin Microbiol Antimicrob.

[6] García-Benítez V, García-Hidalgo L, Archer-Dubon C, Orozco-Topete R (2002). Acute primary superficial cutaneous nocardiosis due to Nocardia brasiliensis: a case report in an immunocompromised patient. Int J Dermatol.

[7] Brown-Elliott BA, Brown JM, Conville PS, Wallace RJ Jr (2006). Clinical and laboratory features of the Nocardia spp. based on current molecular taxonomy. Clin Microbiol Rev.

[8] Couble A, Rodríguez-Nava V, de Montclos MP, Boiron P, Laurent F (2005). Direct detection of Nocardia spp. in clinical samples by a rapid molecular method. J Clin Microbiol.

[9] Wilson JW (2012). Nocardiosis: updates and clinical overview. Mayo Clin Proc.

[10] Ambrosioni J, Lew D, Garbino J (2010). Nocardiosis: updated clinical review and experience at a tertiary center. Infection.

[11] Inamadar AC, Palit A (2003). Primary cutaneous nocardiosis: a case study and review. Indian J Dermatol Venereol Leprol.

[12] Gupta ML, Singh P, Goyal BK, Goyal A, Sharma RD (1982). Bilateral conjunctivitis associated with Nocardia asteroides (a case report). Indian J Ophthalmol.

[13] Shelkovitz-Shilo I, Feinstein A, Trau H, Kaplan B, Sofer E, Schewach-Millet M (1992). Lymphocutaneous nocardiosis due to Nocardia asteroides in a patient with intestinal lymphoma. Int J Dermatol.

[14] Inamadar AC, Palit A (2003). Sporotrichoid pattern of cutaneous nocardiosis. Indian J Dermatol Venereol Leperol.

[15] Fahal A, Mahgoub el S, El Hassan AM, Jacoub AO, Hassan D (2015). Head and neck mycetoma: the mycetoma research centre experience. PLoS Negl Trop Dis.

[16] Mitjà O, Marks M, Bertran L (2017). Integrated control and management of neglected tropical skin diseases. PLoS Negl Trop Dis.

[17] Bakhiet SM, Fahal AH, Musa AM (2018). A holistic approach to the mycetoma management. PLoS Negl Trop Dis.

